# Somatic *ARID1A* mutation stratifies patients with gastric cancer to PD-1 blockade and adjuvant chemotherapy

**DOI:** 10.1007/s00262-022-03326-x

**Published:** 2022-11-12

**Authors:** Yun Gu, Puran Zhang, Jieti Wang, Chao Lin, Hao Liu, He Li, Hongyong He, Ruochen Li, Heng Zhang, Weijuan Zhang

**Affiliations:** 1 NHC Key Laboratory of Glycoconjugate Research, Department of Biochemistry and Molecular Biology, School of Basic Medical Sciences, Fudan University, Shanghai, China; 2 Department of General Surgery, Zhongshan Hospital, Fudan University, Shanghai, 200032 China; 3 Department of Gastric Surgery, Fudan University Shanghai Cancer Center, Shanghai, China; 4 Department of Immunology, School of Basic Medical Sciences, Fudan University, Shanghai, 200032 China

**Keywords:** AT-rich interaction domain 1A, Adjuvant chemotherapy, PD-1 inhibitors, Gastric cancer, Predictive biomarker

## Abstract

**Background:**

AT-rich interaction domain 1A (ARID1A) encodes a vital component of switch/sucrose non-fermentable chromatin-remodeling complex. Given its association with genomic instability, we conducted this study to determine whether *ARID1A* mutation status had an impact on therapeutic responsiveness in gastric cancer (GC), especially combinatory chemo-immunotherapy.

**Methods:**

We retrospectively enrolled a total of 1162 patients from five independent cohorts. ZSHS Cohort and TCGA Cohort were designed to inform chemotherapeutic relevance and immunobiology of *ARID1A*-mutant GC based on tissue samples and sequencing data, respectively. MSKCC Cohort, mGC Cohort, and Melanoma Cohort were utilized to interrogate the predictive efficacy of *ARID1A* mutation to programmed cell death protein 1 (PD-1) blockade.

**Results:**

*ARID1A* mutation was enriched in EBV-positive, hypermutated-single nucleotide variant and microsatellite-unstable subtype GC, and was predictive of responsiveness to both fluorouracil-based chemotherapy and PD-1 blockade. Specifically, *ARID1A* mutation score was a highly sensitive indicator (91%) of response to pembrolizumab. Mechanistically, *ARID1A* mutation correlated with extensive DNA damage repair deficiency and immunogenic tumor microenvironment (TME) featured by elevated activated subsets of CD8^+^ T cells, CD4^+^ T cells, and NK cells. Type 17T helper cells were typically abundant in *ARID1A*-mutant GC and might be a precondition for chemosensitivity conferred by *ARID1A* mutation. Furthermore, *ARID1A* mutation indicated elevated expression of *VEGFA* and *CLDN18*, as well as over-representation of *ERBB2* and *FGFR2* signaling pathway.

**Conclusions:**

*ARID1A*-mutant GC displayed immunogenic TME and might be a candidate for both monotherapy and the combination of frontline chemotherapy and PD-1 blockade.

**Supplementary Information:**

The online version contains supplementary material available at 10.1007/s00262-022-03326-x.

## Introduction

Currently, surgical resection and conventional chemotherapy remain the standard-of-care treatment for gastric cancer (GC) patients [1, 2]. Fortunately, novel pharmaceuticals, including programmed cell death protein 1 (PD-1) inhibitors, HER-2 inhibitors, and anti-angiogenesis agents, have caused a paradigm shift in GC therapeutics [3]. Generally, there is a trend toward precision oncology with biomarker-guided patient stratification and rational combination [4]. While application of chemotherapy plus PD-1 inhibitors has been brought into frontline clinical trials [5], effective biomarkers for those novel combination strategies remain an unmet need.

Only patients with coordinated tumor genomics and immune microenvironment (TME) may gain maximum benefits from either chemotherapy or immunotherapy [6–8]. On the one hand, accumulated genomic instability is a typical outcome of genotoxic chemotherapy, which needs to be recognized and implemented by the host immune system before finally mediating tumor regression [9, 10]. That is, a favorable TME may represent an essential premise for chemotherapeutic benefits [10]. Accordantly, we have previously demonstrated that the abundance of intratumoral IL17-producing cells was related to improved chemotherapeutic efficacy in GC [11]. On the other hand, the pre-existing genomic instability pays back to determine tumor-intrinsic immunogenicity [9, 12]. As the most known molecular identities, microsatellite instability (MSI) and tumor mutational burden (TMB) are even key determinants of response to immune checkpoint blockade (ICB) [13, 14]. However, MSI or TMB alone is not indicative of chemotherapeutic responsiveness [15, 16], or even relates to adverse clinical outcome [17, 18], reflecting the heterogeneity of tumor genomic alterations and the lack of applicable biomarkers for combination therapy. Therefore, a more dedicated genome-level parameter with known immunologic effect may be a better predictor of potential therapeutic effects.

A chromosome is a reservoir where genetic information resides on. Any tiny modification in the chromatin structure may impact on the cell behavior. Assembled by diverse subunits, switch/sucrose non-fermentable (SWI/SNF) chromatin-remodeling complexes take the responsibility to determine the positions of nucleosomes [19, 20]. Genes encoding subunits of SWI/SNF complexes including *ARID1A*, *ARID2* and *PBRM1* are crucial in maintaining genome fidelity and orderly gene expression, whereas they frequently mutate across human malignancies [21]. Among all, *ARID1A* (AT-rich interaction domain 1A) mutation affects around 25% of GC patients [1, 22]. Although mutant (MUT)-*ARID1A* generally promotes tumor progression [23], it also correlates with immune activation [6] and favorable therapeutic sensitivity [24–26]. Specifically, *ARID1A* mutation is predictive of response to immunotherapy in metastatic urothelial carcinoma and even performs better when combined with *CXCL13*, a chemokine for B cell recruitment [7]. Besides, *ARID1A* deficiency is associated with flawed DNA damage repair (DDR) processes [6, 26–29], shaping a vulnerability for DNA damaging agents. Collectively, we sought to uncover the immunological effect and predictive significance of somatic *ARID1A* mutation in GC.

In this study, we found that *ARID1A* mutation predicted improved efficacy of both fluorouracil-based chemotherapy and PD-1 blockade in GC. *ARID1A*-MUT tumors were highly immunogenic and abundant in activated immune infiltrates. More interestingly, Th17 cells functioned as a precondition for chemosensitivity conferred by *ARID1A* mutation. Therefore, by upregulating Th17 infiltration, PD-1 inhibitors might plausibly sensitize previously unresponsive GC to chemotherapy. Conclusively, somatic *ARID1A* mutation should be recognized as a crucial molecular parameter to guide treatment selection, especially for screening GC patients that are potentially responsive to combinatory chemo-immunotherapy.

## Methods

### ZSHS cohort and tissue samples

This study recruited overall 1162 patients from five cohorts (Supplementary Figure S1), included one tissue-sample-based ZSHS Cohort (Supplementary Table S1), and four sequencing-based cohorts. The ZSHS Cohort originally enrolled 496 GC patients under the approvement of the Clinical Research Ethics Committee of Zhongshan Hospital, Fudan University (Shanghai, China; approval number: Y2015-054). Between August 2007 and December 2008, the aforementioned patients, median aged 60 at surgery, underwent radical gastrectomy and standard D2 lymphadenectomy in Zhongshan Hospital. After surgery, resected fresh tissue samples were constructed as tumor microarrays (TMAs, manufactured by Shanghai Outdo Biotech Co, Ltd) as previous described [30]. TMAs were preserved at 4 °C before immunohistochemistry staining. In this study, 55 patients were excluded due to dot loss or unassessable ARID1A staining, whereas other 25 patients were excluded for incomplete clinical information or unknown therapeutic status. Ultimately, 416 patients with basic clinical data, including sex, age, Lauren classification, tumor-node-metastasis (TNM) stage, grade, and utilization of fluorouracil-based adjuvant chemotherapy were eligible for further analysis. Overall survival time was defined as the period from surgery to death. These patients were followed until April 2014 with a 42-month median follow-up time. All patients included in this study were fully informed and signed the written consent.

### Immunohistochemistry (IHC)

IHC assay was conducted to evaluate ARID1A expression pattern and count intratumoral IL17A^+^ cells in TMAs of ZSHS Cohort. Anti-ARID1A antibody (catalog number: ab182560) and anti-IL17A antibody (catalog number: ab189377) used in this study were purchased from *Abcam*. For deparaffinage, TMA slides were first baked in an oven at 60 °C for 6 h and then immersed in three washes of xylene. After that, TMAs were sequentially placed in graded alcohol from 100%, 95%, 85%, to 75% for rehydrating and then dipped into blockers buffer (ZSGB-BIO) to inhibit endogenous peroxidase. For antigen retrieval, slides were immersed in corresponding buffer solution and heated for 3 min as soon as the pressure cooker had reached full pressure. After blocked with 10% goat serum (ZSGB-BIO) at 37 °C for 2 h, the slides were subsequently incubated with prediluted primary antibody (1: 500 for anti-ARID1A antibody, and 1:100 for anti-IL17A antibody) at 4 °C overnight. Subsequent to reaction with HRP-labeled secondary antibody (ZSGB-BIO) for 30 min at 37 °C, slides were incubated with diaminobenzidine for color rendering (DAB, ZSGB-BIO). Next, TMAs were immersed into hematoxylin (Servicebio) for counterstaining. Ultimately, slides were washed in xylene for three times and mounted with coverslip and mounting medium (ZSGB-BIO). Detailed information of antibodies and other reagents used for immunohistochemistry was provided (Supplementary Table S2).

### Evaluation of IHC

IHC images were evaluated by two experienced pathologists under a microscope at high-power field (HPF, × 200), both of whom were blinded to corresponding patient outcome. Evaluation of *ARID1A* mutational status was performed according to several previous publications [31, 32], and the association between *ARID1A* mutation and reduced protein expression (Supplementary Figure S2A). Consequently, positive intranuclear staining of ARID1A in tumor cells was regarded as normal ARID1A expression (Supplementary Figure S2B, left), in corresponding to *ARID1A* wildtype (WT). In contrast, negative intratumoral ARID1A expression with positive staining of stromal cells was defined as deficient ARID1A expression (Supplementary Figure S2B, right), pointing to *ARID1A* mutation. Stromal cells were regarded as internal positive control [32]. Tissue samples with negative staining in both tumor nucleus and stromal cells were regarded as unassessable and excluded from subsequent analysis. Tumors with contradictory disposition in ARID1A expression pattern from the two pathologists would receive a second-round evaluation. The density of intratumoral IL17A^+^ cells were defined as the average number of positive stained cells per HPF in three representative areas. Cut-off value of intratumoral IL17A^+^ cells, which was 46, was defined according to our previous study [11].

### Sequencing data and analysis

Inclusion and exclusion criteria along with key study procedures of the four sequencing-based cohorts were provided (TCGA Cohort, MSKCC Cohort, mGC Cohort and Melanoma Cohort; Supplementary Figure S1 and Supplementary Table S3). Bioinformatic analyses performed in this study were generally based on R-4.0.3 (R Foundation for Statistical Computing, http://www.r-project.org/). All used software and R packages were listed (Supplementary Table S4). Briefly, TCGA (*The Cancer Genome Atlas*) Cohort with clinical, transcriptomic, genomic and pathway activity data were obtained from http://www.cbioportal.org and https://xenabrowser.net/ in August 2020 [33]. Drug information of TCGA Cohort was collected directly from Genomic Data Commons (GDC) with “TCGAbiolinks” R package in April 2021. Originally 440 GC patients with clinical information were enrolled, of whom 135 patients with detailed chemotherapy modality description were defined as “With Chemotherapy.” Nine patients were excluded from subsequent analysis for unknown *ARID1A* mutational status or lack of vital status. Clinical and genomic data of Memorial Sloan-Kettering Cancer Center (MSKCC) Cohort were obtained from http://www.cbioportal.org in August 2021, baseline, treatment and survival data of which were presented in Supplementary Table S5 [34]. Metastatic Gastric Cancer (mGC) Cohort with transcriptomic data and therapeutic response were collected from http://tide.dfci.harvard.edu/download/ in March 2021, baseline and treatment data of which were presented in a published study [35]. Melanoma Cohort with therapeutic status and transcriptomic data were obtained from a published study [36]. For TCGA Cohort and Melanoma Cohort, previously reported 28-immune-cell signature [37] was used to evaluate infiltrates with transcriptomic data. Signature was scored by single sample gene set enrichment analysis (ssGSEA) method with “GSVA” R package. Cut-off value of Th17 cells in TCGA Cohort was automatically defined by X-tile (Version 3.6.1, Yale University). Original source of used signatures was provided (Supplementary Table S6).

### Statistical methods

Chi-square and Fisher’s exact test were conducted with MedCalc Statistical Software version 15.6.1 (MedCalc Software bvba, Ostend, Belgium). Remaining statistical analyses included in this study were performed by SPSS software version 23.0 (IBM SPSS). All conducted analyses were two sided, and *P* < 0.05 was defined as statistically significant. Tests of normality and homogeneity of variance were firstly conducted to describe the overall data distribution. Subsequently, specified statistical models were appropriately chosen as follows. Briefly, continuous variables containing two subgroups were analyzed by Student’s *t* test or Mann–Whitney *U* test. Datasets with three or more subgroups were analyzed by one-way ANOVA subsequent by Tukey’s multiple comparisons test. For categorical variables, Chi-square test or Fisher’s exact test was applied. For variables with survival information, survival curves with univariate (unadjusted) and multivariate (adjusted) Cox proportional hazard regression were applied to discover the clinical significance. Patients with missing data were excluded from each statistical model.

## Results

### ARID1A mutation is predictive of chemotherapeutic responsiveness in gastric cancer

**Fig. 1:**
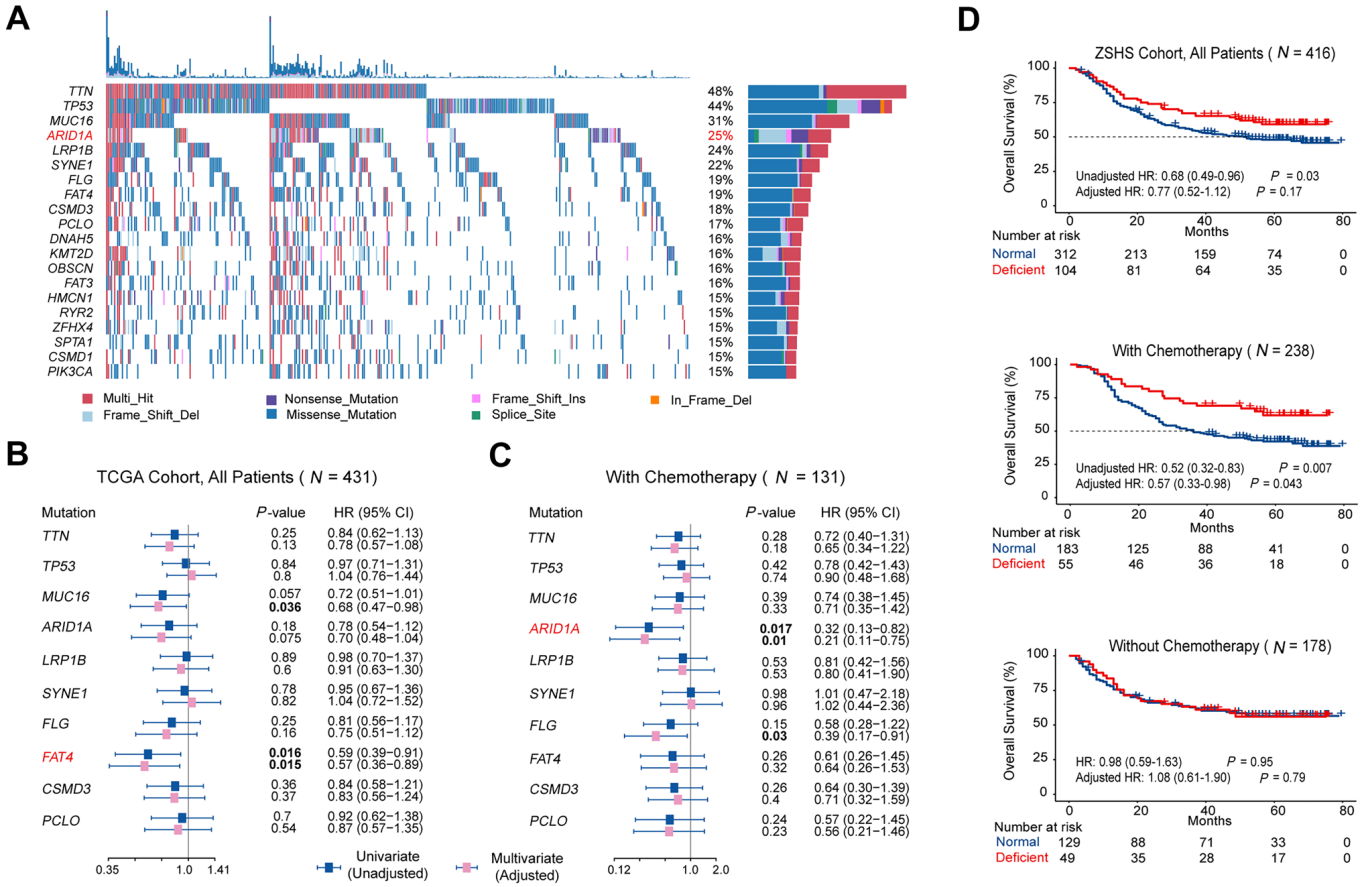
ARID1A mutation predicts favorable chemotherapeutic responsiveness in gastric cancer. **A** Oncoplot showed distribution and alteration pattern of top 20 mutations in TCGA Cohort. **B**, **C** Forest plots profiled the clinical significance regarding overall survival (OS) of 10 most frequently mutated genes in TCGA Cohort. *FAT4* mutation indicated favorable OS in the whole TCGA Cohort (all patients, *N* = 431) which comprised patients with exact chemotherapeutic modalities and unknown chemotherapeutic status (**B**). In patients with exact chemotherapeutic modalities (With Chemotherapy, *N* = 131), *ARID1A* mutation was the only one to inform prolonged OS in both univariate (unadjusted) and multivariate (adjusted) Cox regression model. Multivariate Cox regression model incorporated corresponding mutations and fundamental clinicopathological parameters including age, sex, TNM stage, histology, and grade. **D** In ZSHS Cohort, survival curves displayed the clinical significance of *ARID1A* alteration status regarding OS. Deficient ARID1A indicated favorable OS in all patients regardless of chemotherapy status (Top), patients with fluorouracil-based adjuvant chemotherapy (Middle), but not in those did not receive chemotherapy (Bottom). Multivariate Cox regression model incorporated *ARID1A* and clinicopathological parameters including age, sex, TNM stage, histology, and grade. All presented *P* values were two sided

Existing molecular subtyping of gastric cancer is primarily based on overall genomic features including mutation density and chromosomal instability (CIN) [38, 39]. However, the immunobiology and clinical significance of specific gene alterations were somehow ignored. Therefore, we screened top mutations in the TCGA Cohort to discover potential targetable or predictive alterations (Fig. 1A). For all patients in TCGA Cohort regardless of therapeutic status (All Patients, *N* = 431), *FAT4* mutation was the only one that conferred prolonged overall survival (OS) in both univariate (Fig. 1B, HR: 0.59, *P* = 0.016) and multivariate Cox regression model (Fig. 1C, HR: 0.57, *P* = 0.015). Nevertheless, when merely focused on patients with known chemotherapy information (With Chemotherapy, *N* = 131), the presence of *FAT4* mutation no longer implied a trend toward improved OS.

Instead, *ARID1A*-MUT GC patients showed favorable OS compared with WT tumors when receiving chemotherapy (Fig. 1C and Supplementary Figure S2C, univariate HR: 0.32, *P* = 0.017; multivariate HR: 0.21, *P* = 0.01). IHC staining of ARID1A was performed in our internal ZSHS Cohort to validate this finding (Supplementary Figure S2B). Consistently, deficient ARID1A predicted superior OS in patients with fluorouracil-based adjuvant chemotherapy (Fig. 1D, univariate HR: 0.52, *P* = 0.007; multivariate HR: 0.57, *P* = 0.043), but not in patients who merely received surgical resection (Without Chemotherapy). Exactly, *ARID1A* mutation affected around 25% of GC patients and took the fourth place of top mutated genes. *ARID1A* mutation was generally co-occurrent with other mutations except *TP53*, while frameshift insertion or deletion consisted of a large proportion of *ARID1A* mutants (Supplementary Figure S2D and S2E).

### ARID1A mutation correlates with extensive DDR deficiency and immune activation.

We sought to characterize the underlying biological process affected by *ARID1A* mutation through differential gene expression and pathway analysis. Consequently, *ARID1A-*MUT GC showed a remarkable upregulation in IFN-gamma signaling related genes, including *IFNG*, *CXCL9*, *CXCL10*, *CXCL11* and *CD274* (Supplementary Figure S3A). Meanwhile, pathways reflecting cell cycle activity were also over-represented (Supplementary Figure S3B).

**Fig. 2:**
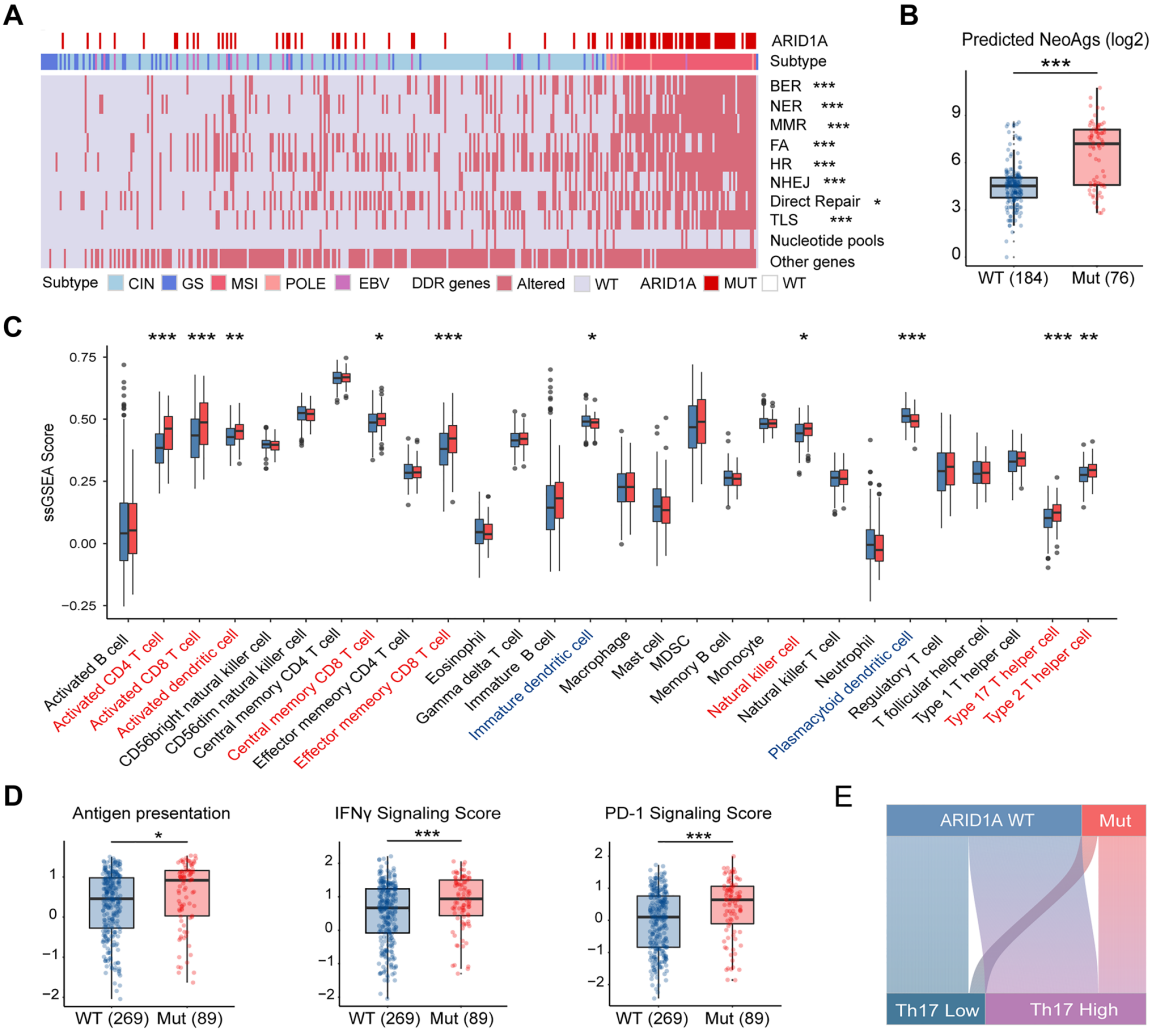
ARID1A mutation fuels immunogenic tumor microenvironment. **A** Heatmap displayed association between *ARID1A* mutation and alterations in DNA damage repair (DDR) genes. Non-silent mutation, deep deletion, or epigenetic silencing in any genes of selected pathway were regarded as alterations in DDR pathway. BER: Base Excision Repair; NER: Nucleotide Excision Repair; MMR: Mismatch Repair; FA: Fanconi Anemia; HDR: Homology-dependent recombination; NHEJ: Non-homologous End Joining; TLS: Translesion Synthesis. **B** Boxplot showed an elevation in predicted neoantigens (NeoAgs) in *ARID1A*-mutant GC tumors. ***P < 0.001. **C** Boxplots profiled 28 subsets of immune infiltrates across *ARID1A* mutational status calculated by ssGSEA method. *P < 0.05, **P < 0.01, ***P < 0.001. Significantly elevated immune infiltrates were marked in red, whereas significantly reduced ones were colored in blue. **D** Boxplots showed representative functional signatures based on *ARID1A* mutational status. *P < 0.05, ***P < 0.001. **E** Sankey diagram displayed association between *ARID1A* mutational status and type 17 T helper cells (Th17) abundance. All presented *P* values were two sided

Combined with the known biology of SWI/SNF complex to maintain genomic stability and enable orderly gene expression, we queried whether *ARID1A* mutation acted on DDR machinery [40]. Herein, we found that *ARID1A* mutants suffered from extensive DDR deficiency, which included the deficiency of base excision repair (BER), nucleotide excision repair (NER), mismatch repair (MMR), homology-dependent recombination (HR), etc. (Fig. 2A), reflecting the failure to maintain a stable genome. Further, *ARID1A*-MUT GC harbored significantly elevated neoantigens predicted by computed method [41] (Fig. 2B), and exhibited an inflamed immune contexture toward elevated antitumor cell components [37] and enhanced immunogenic functionality [33]. Specifically, effector and activated subsets of CD8^+^ T, CD4^+^ T and NK cells were dramatically enriched in *ARID1A*-MUT TME (Fig. 2C). Besides IFN-gamma signaling, PD-1 pathway to inhibit over-reactivity was also elevated, manifesting a trend toward maximal immune activation (Fig. 2D).

### Type 17 T helper cells are accumulated in ARID1A-MUT tumors and indicate chemotherapeutic responsiveness.

Among all functional immune subsets elevated in *ARID1A*-MUT tumors, however, only type 17 T helper cells (Th17) correlated with improved OS in GC patients with chemotherapy (Supplementary Figure S4A, univariate HR: 0.46, *P* = 0.014; multivariate HR: 0.43, *P* = 0.014). Our prior study has uncovered the antitumor role of IL17 producing cells and its relation with chemotherapeutic sensitivity in gastric cancer [11]. Consequently, we herein focused on the interplay between *ARID1A* mutation and Th17 infiltration (Fig. 2E). IHC staining and evaluation of IL17 producing cells were performed in ZSHS Cohort (Supplementary Figure S4B). Consistent with TCGA Cohort, ARID1A-deficient tumors were significantly abundant in intratumoral IL17 producing cells (Supplementary Figure S4C).

### Chemotherapeutic sensitivity conferred by ARID1A mutation requires Th17 abundance.

**Fig. 3:**
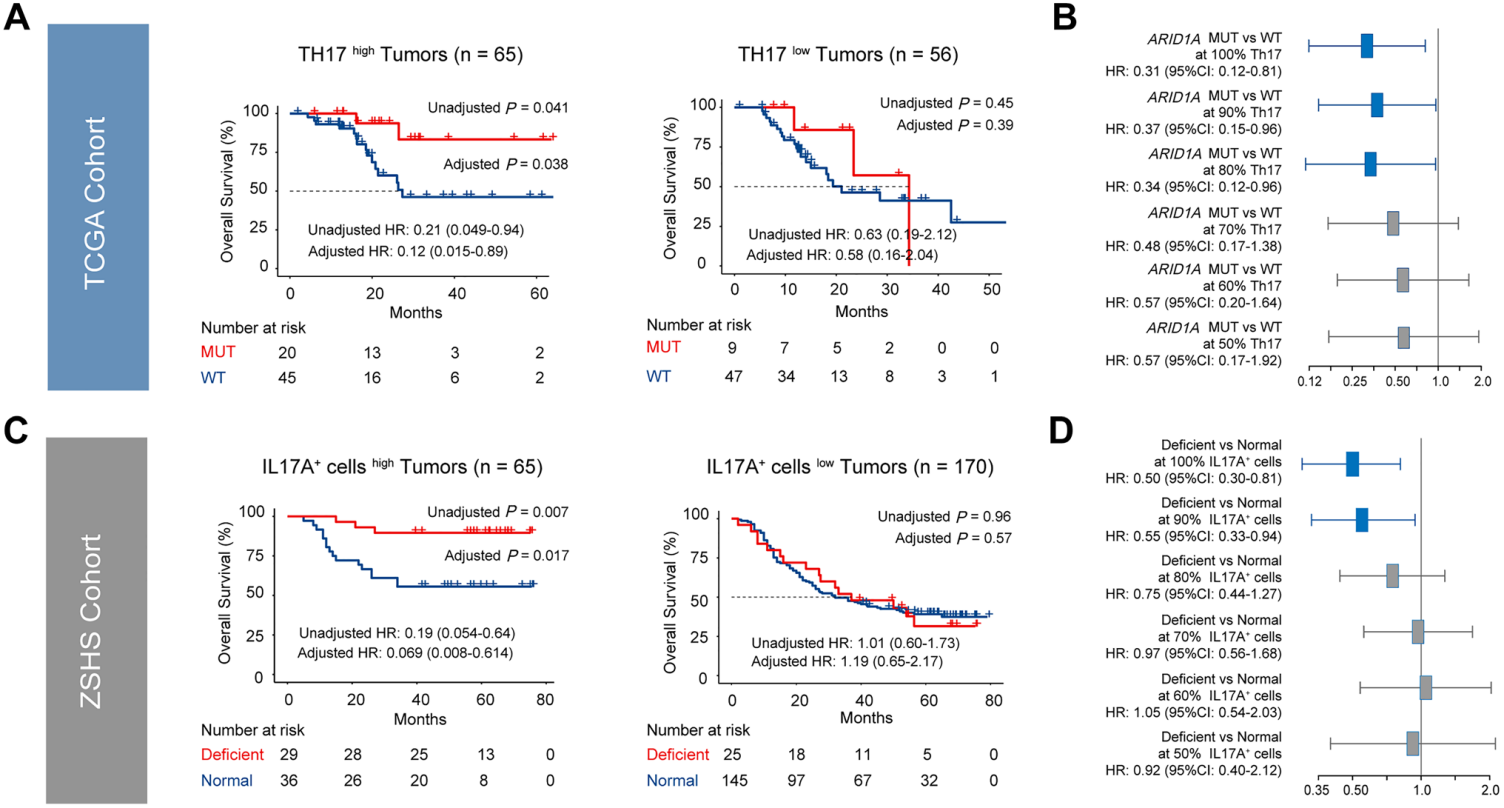
Chemotherapeutic sensitivity by ARID1A mutation requires Th17 infiltration. **A** In TCGA Cohort, survival curves displayed associations between *ARID1A* mutational status and OS in subgroups based on Th17 abundance. *ARID1A* mutation predicted superior OS in Th17^high^ subgroup, but not in Th17^low^ subgroup. **B** Forest plots showed predictive power of *ARID1A* status based on different Th17 cut-offs. When patients held top 30% Th17 were removed, *ARID1A* mutation no longer indicated prolonged OS in TCGA Cohort. **C** In ZSHS Cohort, survival curves displayed associations between ARID1A status and OS in subgroups based on intratumoral IL17A^+^ cells abundance. ARID1A deficiency predicted superior OS in IL17A^+^ cells^high^ subgroup, but not in IL17A^+^ cells^low^ subgroup. **D** Forest plots showed predictive power of ARID1A status based on different Th17 cut-offs. When patients held top 20% IL17A^+^ cells were removed, ARID1A deficiency did not indicate prolonged OS in ZSHS Cohort (**D**)

We sought to elucidate the association between Th17 infiltration and chemotherapeutic sensitivity of *ARID1A*-MUT GC. In TCGA Cohort, predictive role of *ARID1A* mutation based on Th17 infiltration was examined both dichotomously (Fig. 3A) and continuously (Fig. 3B). As a result, *ARID1A* mutation predicted favorable OS in Th17^high^ tumors (Fig. 3A, univariate HR: 0.21, *P* = 0.041; multivariate HR: 0.12, *P* = 0.038), but not in Th17^low^ tumors. Similarly, when patients with top 30% Th17 infiltration were removed, *ARID1A* mutation was no longer predictive of chemotherapeutic sensitivity in GC (Fig. 3B, left), suggesting the dose-dependent effect of Th17 cells. Results from ZSHS Cohort were in line with the above findings (Fig. 3C, left, univariate HR: 0.19, *P* = 0.007, multivariate HR: 0.069, *P* = 0.017; Fig. 3D, Th17 cut-off: 80%). Conclusively, Th17 cells abundance within TME might be a precondition for the chemosensitivity of *ARID1A*-MUT GC. More interestingly, our analysis in Melanoma Cohort suggested that receiving PD-1 blockade (nivolumab) resulted in elevated Th17 infiltration (Supplementary Figure S6), which encouraged us to uncover the linkage between *ARID1A* mutation and response to PD-1 inhibitor.

### AR1D1A mutation indicates response to PD-1 inhibitors and associates with targetable genomic alterations.

**Fig. 4:**
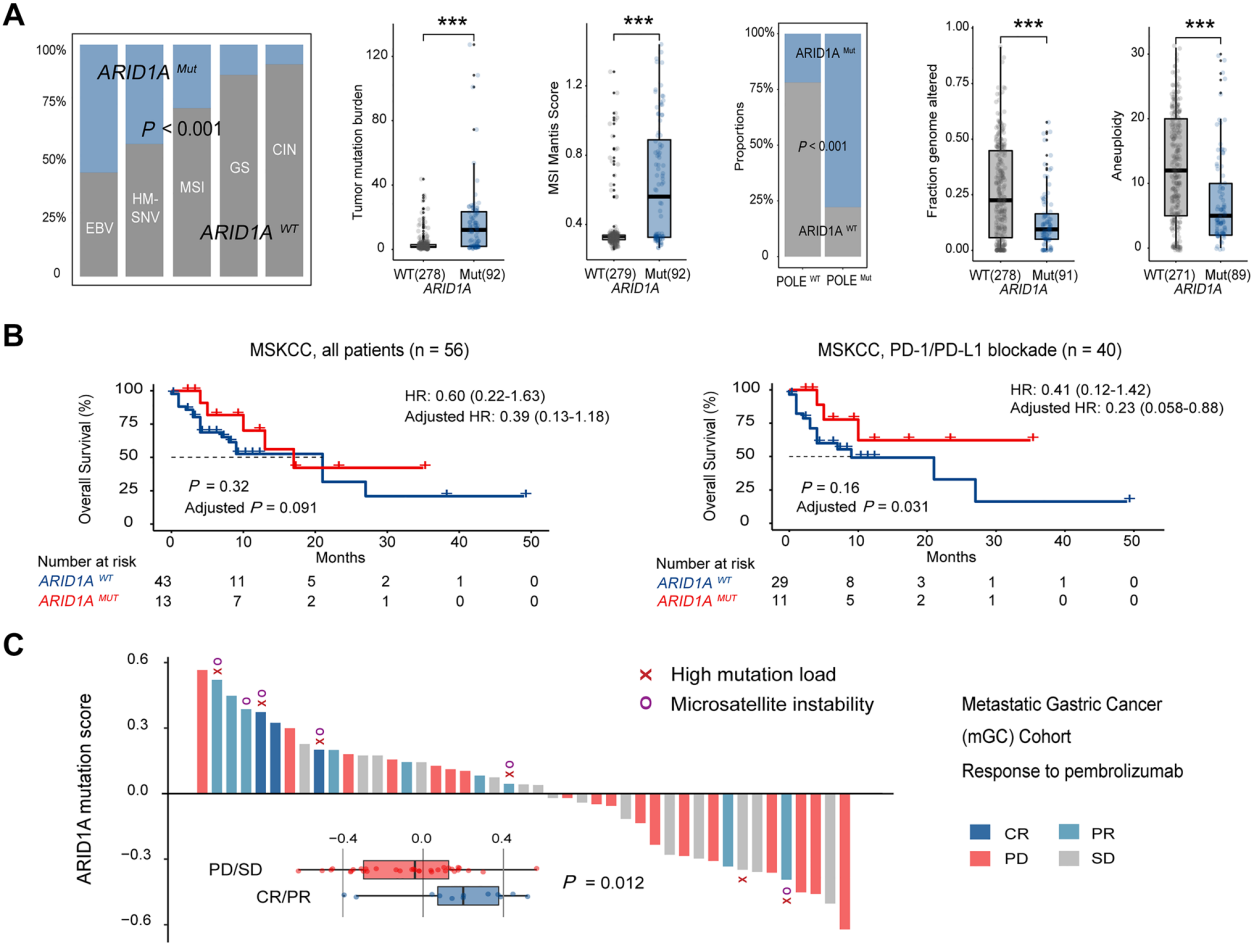
ARID1A mutation shapes superior responsiveness to PD-1 blockade. **A** Bar charts and box plots displayed linkage between *ARID1A* mutation and molecular subtype classifiers. *ARID1A* mutation frequently occurred in EBV-positive, hypermutated-single nucleotide variant (HM-SNV) and microsatellite-unstable (MSI) subtype GCs, with positive association with tumor mutation burden (TMB), MSI Mantis score, and *POLE* mutation. Contrastingly, *ARID1A* mutation negatively correlated with the fraction of altered genome and chromosomal aneuploidy. ***P < 0.001. **B** In MSKCC Cohort, GC patients with *ARID1A* mutation showed a trend toward improved OS when receiving PD-1/PD-L1 inhibitor therapy (Right). However, no significant difference was observed in all patients regardless of therapeutic modalities (Left). **C** In mGC Cohort, bar charts displayed association between *ARID1A* mutation score and immunotherapeutic response. CR: complete response, PR: partial response, SD: stable disease, PD: progressive disease. All shown *P* values were two tailed. Responder: CR or PR

Currently, TCGA subtyping remains one of the most commonly used stratification methods for GC [38]. Herein, *ARID1A* mutations were mainly enriched in EBV-positive (EBV^+^), microsatellite-unstable (MSI), and *POLE*-MUT (hypermutated-single nucleotide variation, HM-SNV) tumors (Fig. 4A). Exactly, *ARID1A* mutants showed a distinctive genomic contexture featured by increased TMB and MSI Mantis score [42], yet reduced fraction genome altered and chromosomal aneuploidy. In MSKCC Cohort, *ARID1A* mutation failed to predict OS among all GC patients when univariate Cox analysis was conducted. However, when merely focused on patients receiving anti-PD-1/PD-L1 monotherapy, *ARID1A* mutation was indicative of prolonged OS in multivariate Cox model after adjusted by age and sex (Adjusted HR: 0.23, *P* = 0.031) (Fig. 4B). To validate this finding in other cohorts that generally lack mutation data, we used TCGA Cohort to construct an “*ARID1A* mutation score” by calculating the Z-score of upregulated genes (Log_2_ FC > 2, *ARID1A* MUT *vs* WT, Supplementary Figure S4D) minus Z-score of downregulated genes (Log_2_ FC < − 2) [43]. Consequently, *ARID1A* mutation score could not only mimic *ARID1A* mutational status with RNA-seq data, but also reflect the functional status of *ARID1A* mutants. In mGC Cohort, when combining *ARID1A* mutation score with TMB and MSI, there was superior sensitivity (91%) to capture ICB responders (Fig. 4C). Eleven out of the total twelve responders held at least one features comprising positive *ARID1A* mutation score, high TMB, or MSI.

Additionally, we assessed the association between *ARID1A* mutation and known targetable alterations [3, 44] (Supplementary Figure S6). As a result, *ARID1A* mutants displayed significantly elevated *VEGFA* expression, *ERBB2* signaling, *FGFR2* signaling, and *CLDN18* expression, yet equal *EGFR* signaling and c-Met signaling to WT GC tumors. Collectively, those findings might provide a roadmap for targeted therapy selection in GC patients.

## Discussion

*ARID1A* mutation has been explored as a predictor of immunotherapeutic responsiveness in metastatic urothelial carcinoma and pan-cancer analysis [7, 45]. However, through integrative analysis of five independent cohorts, we are the first to report *ARID1A*-MUT GC as a molecularly distinct subtype with immunogenic TME and broad therapeutic targets.

Several insights were gained from our research. First, we showed that *ARID1A*-MUT GC patients could benefit from both chemotherapy and PD-1 blockade. *ARID1A* mutation affected almost 25% of GC patients and was the only one to inform chemotherapeutic responsiveness across the top ten mutated genes. This finding strongly encourages us to supplement *ARID1A* mutation as a crucial parameter in future molecular subtyping of GC. Notably, when combining *ARID1A* mutation score with TMB and MSI, 11 out of 12 responders in mGC Cohort were successfully predicted. If confirmed in larger-scaled prospective clinical trials, *ARID1A* mutation score may function as a highly sensitive indicator to screen possible ICB responders.

Second, our study indicated that *ARID1A* mutation might serve as a novel biomarker for chemotherapy plus PD-1 blockade. *ARID1A* mutation was prevalent in all five TCGA molecular subtypes. While MSI subtype GC and *POLE*-MUT patients with high TMB were likely to benefit from immunotherapy, existing evidence suggests they may not be suitable for chemotherapy [15–18]. Therefore, stratifying GC patients by *ARID1A* mutational status might be a solid mean to enable beneficial chemo-immunotherapy. Moreover, given PD-1 blockade resulted in elevated Th17 infiltration, prior immunotherapy to shape a “chemotherapy friendly” TME might augment chemotherapeutic benefits for *ARID1A*-MUT patients. Thus, our study even implied the feasibility of executing PD-1 blockade—chemotherapy sequential treatment in *ARID1A*-MUT GC patients.

Third, we profiled an “*ARID1A* mutation—DDR deficiency—immune activation” axis in GC and provided a feasible explanation for chemosensitivity conferred by genomic alterations. Intratumoral immune infiltrates are key determinants of patient outcome and therapeutic vulnerability [10]. We have previously demonstrated the antitumor role of intratumoral IL17 producing cells [11], which were dominated by Th17 cells. The current study further specified that Th17 abundance could even function as the basis for chemotherapeutic vulnerability conferred by *ARID1A* mutation. Although thoroughly elucidating chemotherapeutic effect remains a challenge, our results again recognized immune cells as key players, and underscored the interplay between tumor genomics and immune contexture in determining patient outcomes. Moreover, given *ARID1A*-MUT held a highly unstable genome, ATR inhibitors [25] and PARP inhibitors [26] targeting DNA repair pathways might potentially cooperate with chemo-immunotherapy in overcoming therapeutic resistance.

Limitations were presented. First, our results were primarily based on observations of retrospective cohorts. Moreover, the safety of combinatory therapy for *ARID1A*-MUT GC was not assessed. Therefore, our suggestion of *ARID1A* mutation as a biomarker for multimodality treatment needs to be validated in preclinical experiments or prospective clinical trials.

## Supplementary Information

Below is the link to the electronic supplementary material.

Supplementary file 1 (DOC 13260 kb)

## Data Availability

Data and materials generated that are relevant to the results are included in this article. Other data are available from the corresponding author Prof. Xu upon reasonable request.

## Abbreviations

ARID1A: AT-rich interaction domain 1A
BER: Base excision repair
CIN: Chromosomal instability
DDR: DNA damage repair
GC: Gastric cancer
HM-SNV: Hypermutated-single nucleotide variant
HR: Homology-dependent recombination
ICB: Immune checkpoint blockade
IHC: Immunohistochemistry
mGC: Metastatic gastric cancer
MMR: Mismatch repair
MSI: Microsatellite unstable
MSKCC: Memorial Sloan-Kettering Cancer Center
MUT: Mutant
NER: Nucleotide excision repair
OS: Overall survival
PD-1: Programmed cell death protein 1
ssGSEA: Single sample gene set enrichment analysis
SWI/SNF: Switch/sucrose non-fermentable
TCGA: The Cancer Genome Atlas
Th17: Type 17T helper cells
TNM: Tumor-node-metastasis
TMAs: Tumor microarrays
TMB: Tumor mutational burden
TME: Tumor microenvironment
WT: Wildtype
