# Supplementary material for: Somatic ARID1A mutation stratifies patients with gastric cancer to PD-1 blockade and adjuvant chemotherapy
Source: Cancer Immunol Immunother. 2022 Nov 12;72(5):1199–208. doi: 10.1007/s00262-022-03326-x (PMC10110689; doi:10.1007/s00262-022-03326-x)
Supplement: Supplementary file 1 — (DOC 13260 kb) [file 262_2022_3326_MOESM1_ESM.doc]

**Somatic *ARID1A* mutation stratifies patients with gastric cancer to PD-1 blockade and adjuvant chemotherapy**

**Running title**: Somatic *ARID1A* mutation in GC

**Keywords**: AT-rich interaction domain 1A; Adjuvant Chemotherapy; PD-1 inhibitors; Gastric cancer; Predictive biomarker

**Supplementary Figures and Tables**

Supplementary Figure S1. Study design.

Supplementary Figure S2. Identification of ARID1A mutation in gastric cancer.

Supplementary Figure S3. ARID1A mutation correlates with cell cycle alteration and immune activation.

Supplementary Figure S4. Association between chemotherapeutic sensitivity and immune infiltrates enriched in ARID1A mutant GC.

Supplementary Figure S5. PD-1 blockade unleashes TH17 infiltration in melanoma.

Supplementary Figure S6. Association between ARID1A mutation status and targetable alterations.

Supplementary Table S1. Patient demographics of ZSHS Cohort.

Supplementary Table S2. Antibodies and reagents used for IHC.

Supplementary Table S3. Patient demographics of TCGA Cohort.

Supplementary Table S4. Software and package availability.

Supplementary Table S5. Patient demographics of MSKCC Cohort.

Supplementary Table S6. Signature availability.


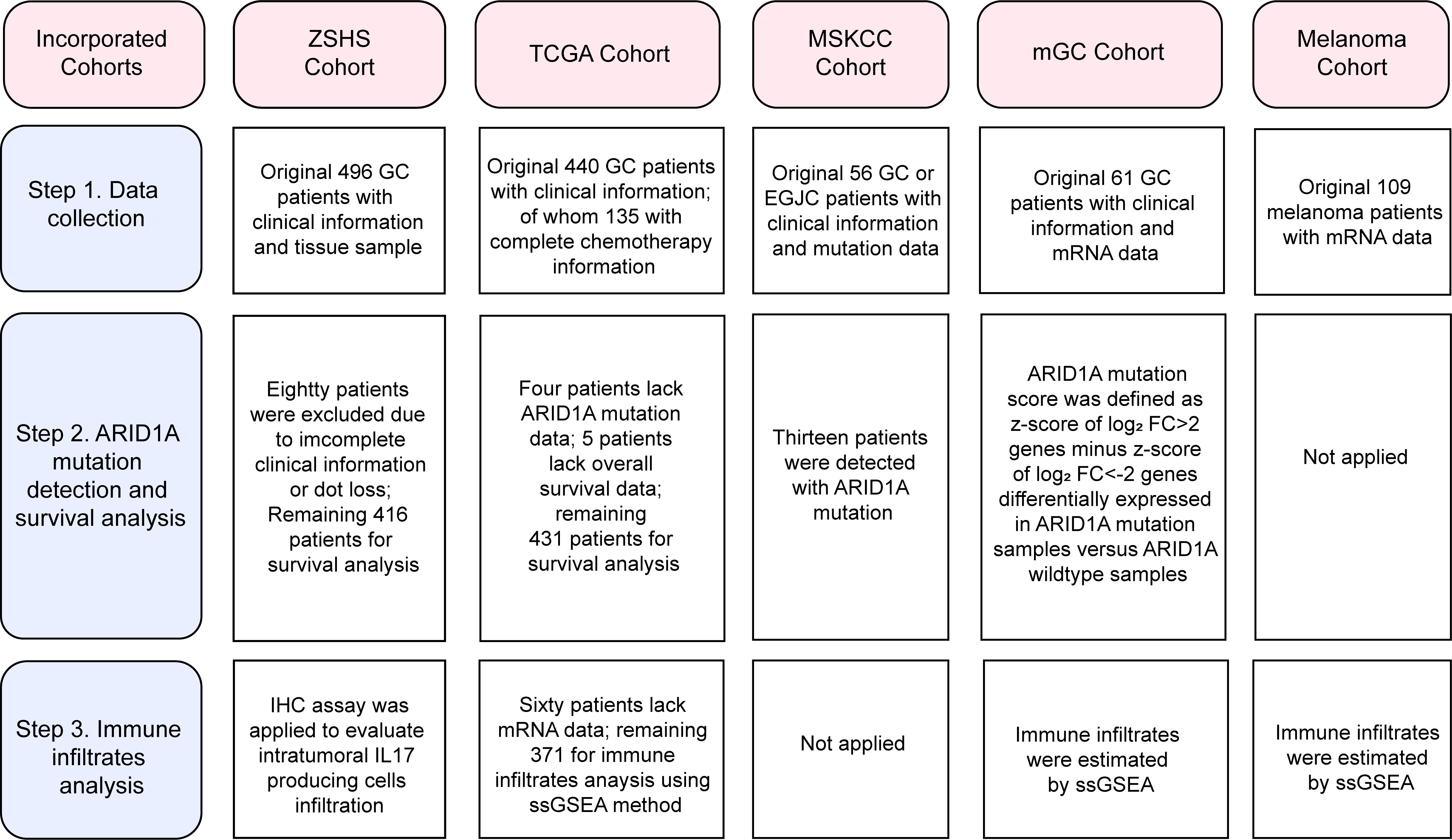


**Supplementary Figure 1. Study design.**

Abbreviations: ZSHS Cohort (Zhongshan hospital Cohort), TCGA Cohort (*The Cancer Genome Atlas* Cohort), MSKCC Cohort (Memorial Kettering-Sloan Cancer Center Cohort), mGC Cohort (Metastatic Gastric Cancer Cohort), GC (gastric cancer), EGJC (esophagogastric junction cancer), FC (fold change), ssGSEA (single sample gene set enrichment analysis).


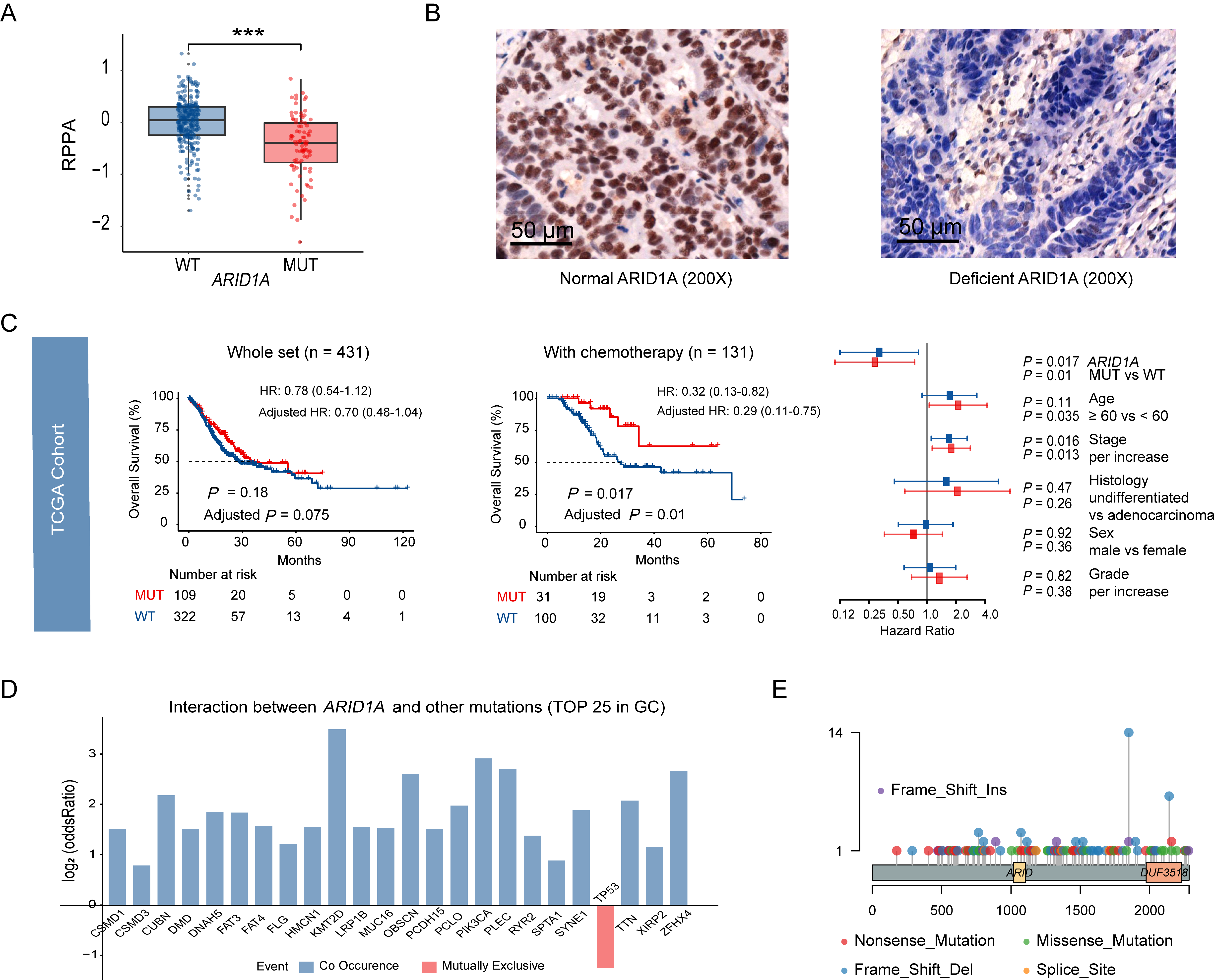


**Supplementary Figure 2. Identification of *ARID1A* mutation in gastric cancer.**

(A) *ARID1A* mutant GC held significantly reduced ARID1A protein expression. RPPA: reverse-phase protein arrays. (B) Representative IHC staining of ARID1A in TMAs of ZSHS Cohort. Positive cells were stained in brown. Original magnification: 200X. (C) Survival curves showed the prognostic role of *ARID1A* mutation in TCGA Cohort and the multivariate Cox regression model. (D) *ARID1A* mutation co-existed with other top mutations in GC except TP53. (E) Mutation patterns and positions of *ARID1A* in TCGA Cohort.


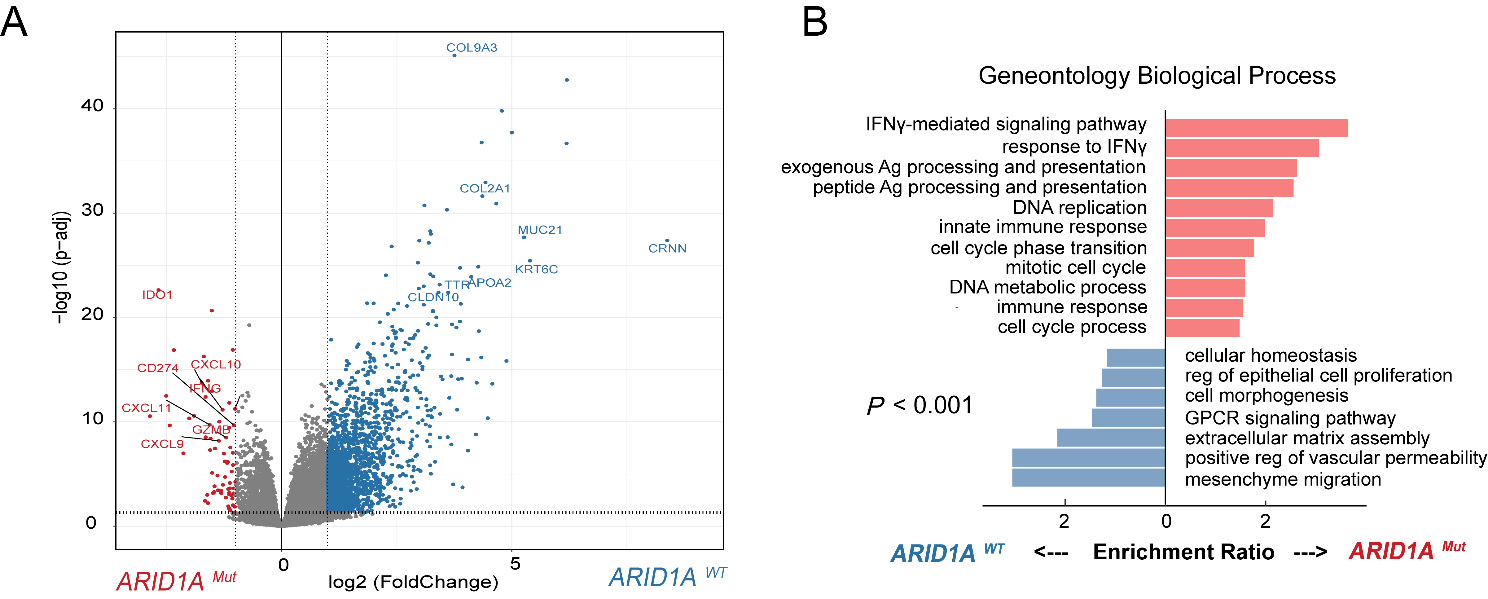


**Supplementary Figure 3. *ARID1A* mutation correlates with cell cycle alteration and immune activation.**

(A) Volcano plot displayed differentially expressed genes (DEGs) in *ARID1A* mutant GC compared with wildtype tumors. (B) Barplot showed pathways mapped by DEGs based on *ARID1A* mutation status. All presented *P* < 0.001. Ag: antigen, reg: regulation.


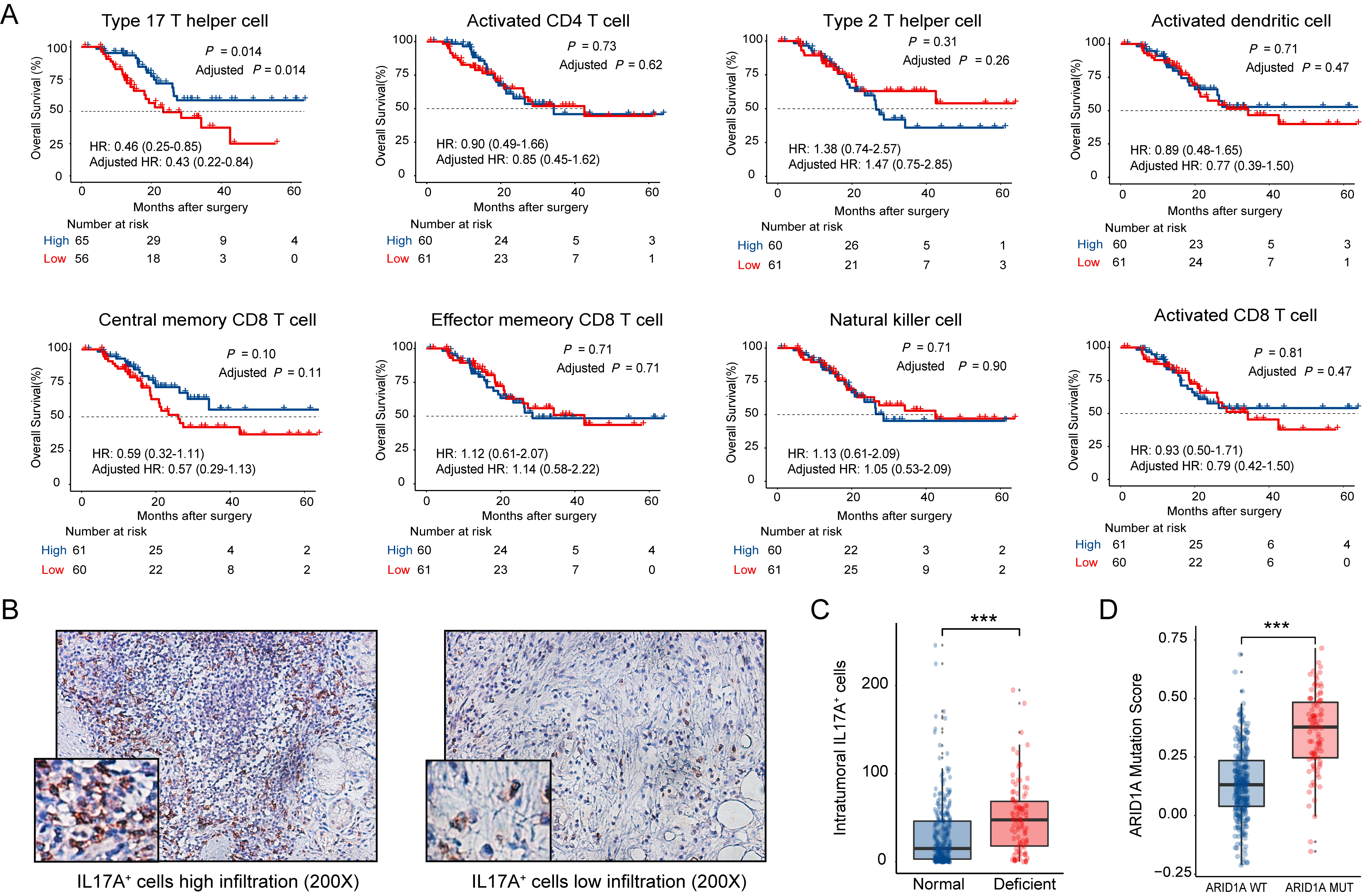


**Supplementary Figure 4. Association between chemotherapeutic sensitivity and immune infiltrates enriched in *ARID1A* mutant GC.**

(A) Survival curves displayed the prognostic efficacy of immune infiltrates enriched in *ARID1A* mutant tumors in patients with chemotherapy of TCGA Cohort. (B) Representative IHC staining of IL17A^+^ cells in TMAs of ZSHS Cohort. IL17A^+^ cells were stained in brown. Original magnification: 200X. (C) In ZSHS Cohort, tumors with aberrant *ARID1A* staining held significantly elevated IL17A^+^ cells infiltration. (D) Boxplot showed distinctive *ARID1A* mutation score between *ARID1A* wildtype and mutant GC.


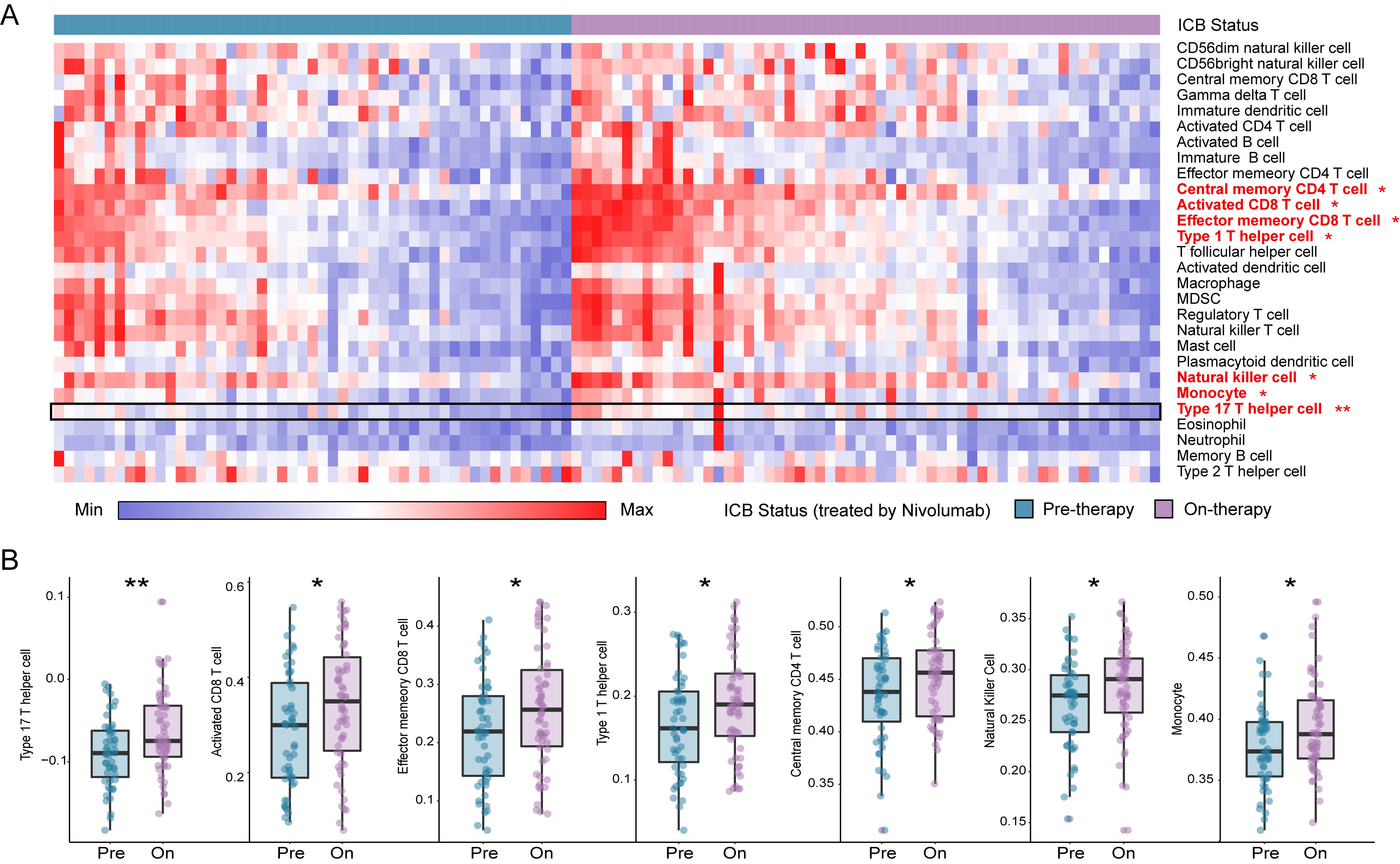


**Supplementary Figure 5. PD-1 blockade unleashes TH17 infiltration in melanoma.**

(A-B) In Melanoma Cohort, immune subsets including Th17 cells, activated CD8 T cells, effector memory CD8 T cells, Th1 cells, central memory CD4 T cells, NK cells and monocytes were elevated after receiving PD-1 blockade (Nivolumab).


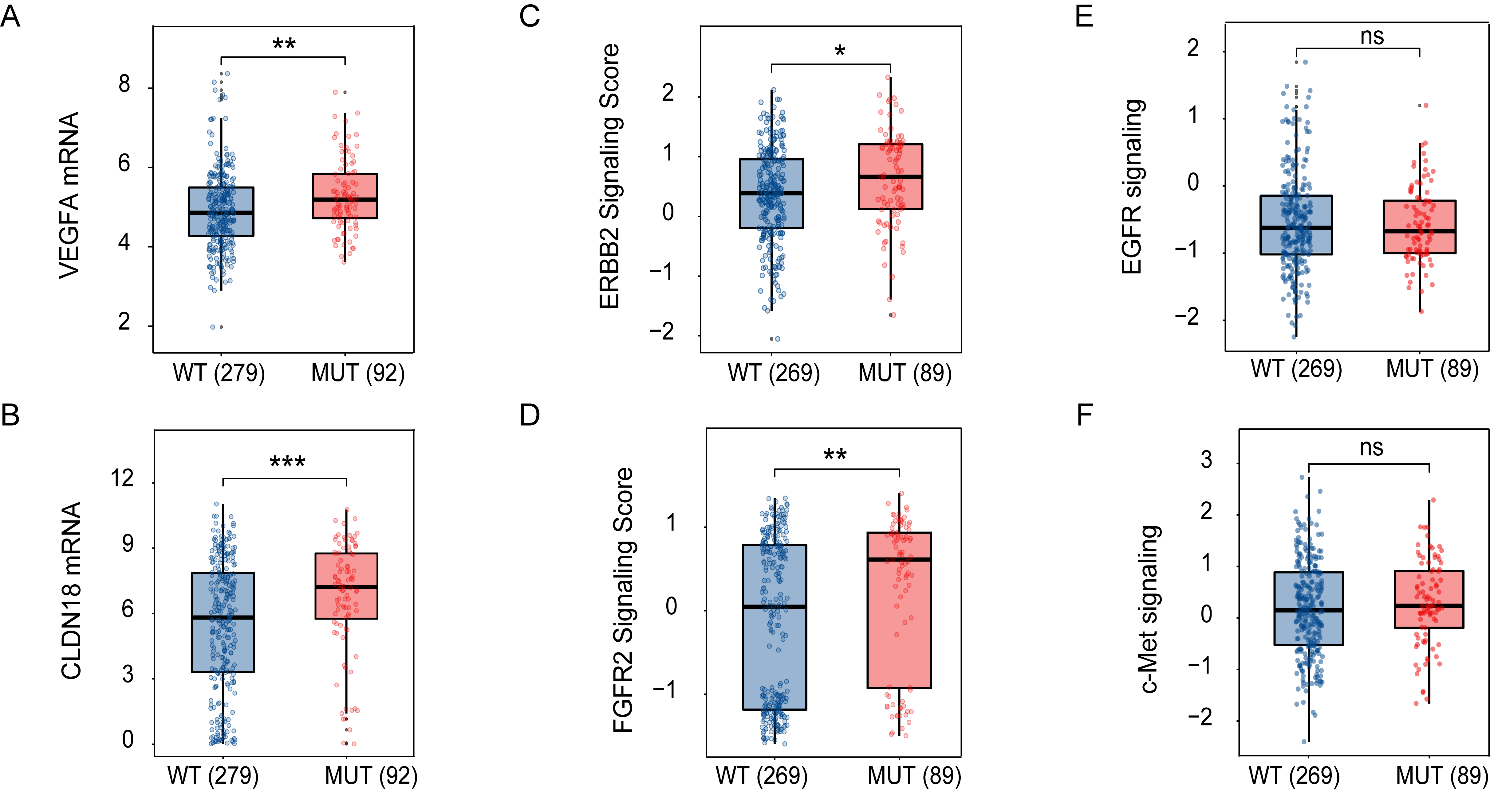


**Supplementary Figure 6. Association between *ARID1A* mutation status and targetable alterations.**

(A-B) *ARID1A* mutant GC held significantly higher VEGFA and CLDN18 expression. (C-D) Signaling score of ERBB2 and FGFR2 were elevated in GC with *ARID1A* mutation. (E-F) No significant difference in EGFR or c-Met signaling was observed between subgroups based on *ARID1A* mutation status. *: *P* < 0.05, **: *P* < 0.01, ***: *P* < 0.001, ns referred to not statistically significant.

**Supplementary Table S1. Patient demographics of ZSHS Cohort.**

| **Factors** |  | **Aberrant ARID1A** | | **Normal ARID1A** | | ***P* value** |
| --- | --- | --- | --- | --- | --- | --- |
| All patients |  | 104 | 100% | 312 | 100% |  |
| Age | ≥ 60 | 51 | 49.04% | 156 | 50.00% | 0.87 |
|  | < 60 | 53 | 50.96% | 156 | 50.00% |  |
| TNM stage | I | 29 | 27.88% | 66 | 21.15% | 0.32 ^a^ |
|  | II | 24 | 23.08% | 65 | 20.83% |  |
|  | III | 47 | 45.19% | 168 | 53.85% |  |
|  | IV | 1 | 0.01% | 7 | 2.24% |  |
|  | Unknown | 3 |  | 6 |  |  |
| Lauren classification | Diffuse | 20 | 19.23% | 80 | 25.64% | 0.39 |
|  | Intestinal | 68 | 65.38% | 192 | 61.53% |  |
|  | Mixed type | 16 | 15.38% | 40 | 12.82% |  |
| Sex | Male | 76 | 73.08% | 218 | 69.87% | 0.53 |
|  | Female | 28 | 26.92% | 94 | 30.13% |  |
| Grade | 1 | 9 | 8.65% | 12 | 3.85% | 0.19 |
|  | 2 | 21 | 20.19% | 59 | 18.91% |  |
|  | 3 | 73 | 70.19% | 240 | 76.92% |  |
|  | 4 | 1 | 3.85% | 1 | 0.32% |  |
| Adjuvant chemotherapy ^b^ | Applied | 55 | 52.88% | 183 | 58.65% | 0.30 |
|  | Not applied | 49 | 47.12% | 129 | 41.35% |  |
| Vital status | Alive | 63 | 60.58% | 152 | 48.72% | **0.036 ^c^** |
|  | Deceased | 41 | 39.42% | 160 | 51.28% |  |
| ^a^ Patients with missing data were excluded from Chi-square or Fisher’s exact test.  ^b^ After surgery, fluorouracil-based adjuvant chemotherapy was primarily given to patients with advanced tumors (stage II/III). Patients with adjuvant chemotherapy received at least 1 cycle of fluorouracil-based chemotherapy. No radiotherapy was administered to anyone of the patients enrolled.  ^c^ *P* < 0.05 were marked in bold. | | | | | | |

**Supplementary Table S2. Antibodies and reagents used for IHC.**

| **Antibody or Reagent** | **Source** | **Dilution** | **Catalog No.** |
| --- | --- | --- | --- |
| Rabbit monoclonal [EPR13501] to ARID1A | Abcam | 1:500 | ab182560 |
| Mouse monoclonal [4K5F6] to IL-17A | Abcam | 1:100 | ab189377 |
| Endogenous peroxidase blockers | ZSGB-BIO |  | PV-9000 (reagent 1) |
| Reaction enhancing solution | ZSGB-BIO |  | PV-9000 (reagent 2) |
| Enhanced HRP-conjugated goat anti-mouse/rabbit IgG polymer | ZSGB-BIO |  | PV-9000 (reagent 3) |
| DAB chromogen (20×) and DAB substrate | ZSGB-BIO |  | ZLI-9019 |
| Goat serum | ZSGB-BIO |  | ZLI-9056 |
| Antigen retrieval buffer | ZSGB-BIO |  | ZLI-9064 |
| Mounting medium | ZSGB-BIO |  | ZLI-9550 |
| TBS buffer | Servicebio |  | G0001-2L |
| HE staining reagent | Servicebio |  | G1005-100ML |

**Supplementary Table S3. Patient demographics of TCGA Cohort.**

| **Factors** |  | **ARID1A mutant** | | **ARID1A wildtype** | | ***P* value** |
| --- | --- | --- | --- | --- | --- | --- |
| All patients |  | 110 | 100% | 326 | 100% |  |
| Age | ≥ 60 | 86 | 78.18% | 216 | 66.26% | **0.018 ^a, b^** |
|  | < 60 | 23 | 20.91% | 107 | 32.82% |  |
|  | Unknown ^b^ | 1 |  | 3 |  |  |
| TNM stage | I | 19 | 17.27% | 38 | 11.66% | 0.41 |
|  | II | 31 | 28.18% | 100 | 30.67% |  |
|  | III | 42 | 38.18% | 145 | 44.48% |  |
|  | IV | 11 | 10.00% | 32 | 9.82% |  |
|  | Unknown | 7 |  | 11 |  |  |
| Histology | Undifferentiated | 2 | 1.82% | 11 | 3.37% | 0.53 |
|  | Adenocarcinoma | 108 | 98.18% | 315 | 96.63% |  |
| Sex | Male | 64 | 58.18% | 216 | 66.26% | 0.13 |
|  | Female | 46 | 41.82% | 110 | 33.74% |  |
| Grade | 1 | 2 | 1.82% | 10 | 3.07% | **0.03** |
|  | 2 | 29 | 26.36% | 126 | 38.65% |  |
|  | 3 | 78 | 70.91% | 182 | 55.83% |  |
|  | Unknown | 1 |  | 8 |  |  |
| Chemotherapy ^c^ | Applied | 31 | 28.18% | 102 | 31.29% | 0.54 |
|  | Unknown | 79 | 71.82% | 224 | 68.71% |  |
| Vital status | Alive | 71 | 64.55% | 193 | 59.20% | 0.34 |
|  | Deceased | 38 | 34.55% | 129 | 39.57% |  |
|  | Unknown | 1 |  | 4 |  |  |
| ^a^ *P* < 0.05 were marked in bold.  ^b^ Patients with unknown data were excluded from Chi-square or Fisher’s exact test.  ^c^ Patients with accurate chemotherapy information were regarded as chemotherapy applied (with chemotherapy), those patients mainly received fluorouracil-based or platinum-based regimen. Remaining patients were regarded as unknown chemotherapy status. | | | | | | |

**Supplementary Table S4. Software and package availability.**

| **Software** | **Provider or Repository** | **Usage** |
| --- | --- | --- |
| SPSS software version 23.0 | IBM SPSS | Statistical analyses including t-test, non-parameter test, Cox regression, and interaction test |
| MedCalc Statistical Software version 15.6.1 | MedCalc Software bvba, Ostend, Belgium | Chi-square test and Fisher’s exact test |
| Morpheus | https://software.broadinstitute.org/morpheus | Drawing heatmaps |
| X-tile, Version 3.6.1 | Yale University | Finding optimal cut-off value for continuous variable |
| Webgestalt |  | Over-representation analysis of differentially expressed genes |
| R-4.0.3 | R Foundation for Statistical Computing | Data visualization |
| R Packages | CRAN or Bioconductor | Data visualization |
| ggplot2, version 3.3.5 | CRAN | Obtaining boxplots and bar charts |
| Maftools, version 2.8.0 | CRAN | Analyzing and visualizing mutation data |
| Survminer, version 0.4.9 | CRAN | Drawing survival curves |
| GSVA, version 1.40.1 | Bioconductor | Calculating ssGSEA score or Z-score based on RNAseq data |
| DESeq2, version 1.32.0 | Bioconductor | Performing differential gene expression analysis |
| TCGAbiolinks | Bioconductor | Requesting and download datasets from GDC |
| ClusterProfiler, version 4.0.2 | Bioconductor | Gene name conversion |

**Supplementary Table S5. Patient demographics of MSKCC Cohort.**

|  |  |
| --- | --- |
| **Characteristics** | **Total (*n* = 56)** |
| Age, median (range), yr | 62 (23-87) |
| Sex |  |
| Male | 42 (75.0%) |
| Female | 14 (25.0%) |
| Race |  |
| Asian | 2 (3.6%) |
| Black or African American | 5 (8.9%) |
| White | 25 (44.6%) |
| NA | 24 (42.9%) |
| ECOG performance status |  |
| 0-1 | 33 (58.9%) |
| 2-3 | 2 (3.6%) |
| NA | 21 (37.5) |
| Cancer Type |  |
| Stomach adenocarcinoma | 35 (62.5%) |
| Gastroesophageal junction adenocarcinoma | 21 (37.5%) |
| Received ICB after 1^st^ line treatment |  |
| Yes | 28 (50.0%) |
| No | 7 (12.5%) |
| NA | 21 (37.5%) |
| Drug Type |  |
| Anti-PD-1/PD-L1 | 40 (71.4%) |
| Anti-CTLA-4 | 1 (1.8%) |
| Combination | 15 (26.8%) |
| Survival, median (95% CI), mo |  |
| Overall | 17.0 (8.5-25.4) |

**Supplementary Table S6. Signature availability.**

| **Signature** | **Original source** |
| --- | --- |
| 28-subset immune infiltrates | Reference No. 37, Charoentong P, Finotello F, Angelova M et al. |
| Alterations in DDR pathways | Reference No. 19, Dutta A, Sardiu M, Gogol M et al. |
| Targetable pathway alterations (ERBB2 signaling pathway, EGFR signaling pathway, FGFR2 signaling pathway, and c-Met signaling pathway) | Reference No. 33, Hoadley KA, Yau C, Hinoue T et al. |
| Predicted neoantigens (NeoAgs) | Reference No. 41, Rooney MS, Shukla SA, Wu CJ et al. |
